# The hidden epidemic of alcohol-induced neurological and psychiatric mortality in the U. S. (1999–2023): trends and disparities

**DOI:** 10.3389/fpubh.2025.1712253

**Published:** 2026-01-08

**Authors:** Ying Liu, Yong Chen, Xueling Xiao, Yuhua Li, Kesheng Hu

**Affiliations:** 1 Department of Cardiac Surgery, YueBei People’ s Hospital, Shaoguan, China; 2 Department of Vascular Surgery, YueBei People’s Hospital, Shaoguan, China; 3 Department of Lab Medicine, Armed Police Corps Hospital of Guangdong Province, Guangzhou, China

**Keywords:** alcohol-induced neurological and psychiatric disorders, CDC WONDER, female, middle-aged and older adults, mortality, place of death, urbanization, White

## Abstract

**Background:**

Alcohol use posed a significant burden to public health, contributing substantially to mortality from neurological and psychiatric disorders. Understanding trends and disparities in alcohol-induced neurological and psychiatric (AINP) mortality is crucial for informed policy and targeted interventions.

**Objectives:**

This study is to analyze trends in AINP mortality and associated demographic and geographic disparities in the US between 1999 and 2023.

**Methods:**

Data from the CDC WONDER database were collected to identify AINP related deaths via ICD-10 codes (F10, G31.2, G62.1). Age-adjusted mortality rates (AAMRs, per 100,000 population) and annual percent changes (APCs) with 95% confidence intervals (CIs) were calculated to assess temporal trends. An autoregressive integrated moving average model was used to predict mortality rate changes in high-risk populations by 2030.

**Results:**

A total of 226,785 AINP deaths were reported. AAMR increased from 2.28 (1999) to 4.17 (2023), with no significant change (1999–2017: APC = −0.04%, *p* = 0.934), accelerated increase (2018–2021: APC = 18.35%, *p* = 0.036), followed by a modest decline (2021–2023: APC = −3.80%, *p* < 0.046). Female AAMR showed a higher APC (3.89%, *p* < 0.001) than males (2.55%). The Midwest showed the steepest APC (3.77%, p < 0.001), while the South and West showed the smallest increases (APC = 1.80 and 2.08%, both *p* < 0.01). Mortality growth rates rosed with increasing urbanization. American Indian/Alaska Native (AI/AN) populations had the highest AAMR, peaking at 21.13 in 2021 (APC 2.50%, *p* = 0.004). White people accounted for most deaths (75.69%), with AMMR increasing continuously (APC = 3.45%, *p* < 0.001)). Mortality peaked at ages 45–54 (crude rate: 7.86/100,000, 26.86% of deaths), 55–64 s most affected (30.98% of deaths). 56.85% of deaths occurred at home and 1.13% were pre-hospital. Female projections show a rising trend (APC 3.89%), while adults aged 45–74 show a slight decrease to 9.50 by 2030 despite a historical APC of 2.81%. Conclusions: From 1999 to 2023, US AINP mortality rosed steadily, disproportionately affecting females, AI/AN and White populations, Midwestern residents, and middle-aged and older adults. Most deaths occurred at home, reflecting healthcare access gaps. Targeted interventions for high-risk groups and regions, along with optimized medical resources allocation, are urgently needed.

## 1 Introduction

Alcohol use disorder and its associated neurological and psychiatric complications have become a major challenge in global public health. In the United States, alcohol-related deaths have raised doubled since 1999, reaching a historic peak of 21.6 per 100,000 people in 2020 (1). It has been reported that alcoholic liver disease was the main chronic cause of alcohol-related deaths overall. However, mortality due to alcohol-induced neurological and psychiatric disorders (AINP) was also an important cause of alcohol-related deaths (2, 3), and reports on this issue were rare.

From a pathophysiological perspective, AINP was associated with neurological phenotypes such as alcohol-induced cognitive impairment (4), alcohol-induced peripheral neuropathy (5), and degenerative diseases (6), as well as psychological traits such as anxiety and depression (7, 8). Changes in neuroplasticity were induced by chronic alcohol consumption, resulting in AINP (9). The process was primarily characterized by an imbalance in the gamma-aminobutyric acid (GABA)-glutamate system. Acute alcohol exposure has been demonstrated an enhancement of GABA inhibition, resulting in the induction of euphoria. However, chronic exposure resulted in receptor down-regulation and increased glutamate excitability. The consequence of this disruption was the impaired prefrontal executive function, whilst the reward circuits within the nucleus accumbens and amygdala were reinforced, thus driving compulsive drinking behaviour (10). Furthermore, at the level of the peripheral nerve, alcohol has been revealed to induce chronic alcoholic polyneuropathy through the process of small fibre degeneration, with the presentation of sensory abnormalities and ataxia (5). In the context of chronic pain, depression is exacerbated by specific neural circuits, notably a hippocampus-prefrontal pathway involving CRH neurons (11). Concurrently, inflammation responses and cortisol secretion could be induced by emotional distress, thereby establishing a self-perpetuating cycle in which pain, inflammation and depression reinforce each other. Clinical trials have showed that targeting inflammatory pathways can alleviate depression in individuals with high baseline inflammation (12). Clinically, Wernicke’s encephalopathy and alcoholic dementia represented the interaction between neurodegeneration and mental abnormalities. Alcohol also resulted impulse control impairment, impaired judgment and perception of reality, and an increased risk of violence (13).

Existing research lacked an in-depth exploration of the long-term epidemiological characteristics of AINP and the dynamic evolution of multidimensional differences, such as those related to gender, race, and region. This discrepancy has resulted in delays in identifying high-risk populations and implementing precise intervention strategies. Although AINP may be preventable, factors such as healthcare disparities, lifestyle choices, and socioeconomic status often adversely affect vulnerable populations (14). In view of the magnitude and significance of the issue, it was imperative to investigate the long-term trends in AINP mortality in order to evaluate the efficacy of current measures and develop future public health strategies.

This study systematically evaluated the spatiotemporal trends in mortality rates among AINP populations by integrating national death registration data from the Centers for Disease Control and Prevention (CDC) Wide-Ranging Online Data for Epidemiologic Research (WONDER) epidemiological database spanning 1999 to 2023. Using age-adjusted mortality rates (AAMR) and annual percentage changes (APCs), the study identified prominent variation associated with gender, race, and geographic region. The findings emphasize critical deficiencies in healthcare systems and underscore pivotal approaches to enhance equitable access to home and community care, such as addressing inequities in health interventions and implementing targeted policies for vulnerable groups. These insights are of great significance in the fight against the social determinants of AINP deaths and the advancement of health equity through targeted strategies. Additionally, the study makes two contributions, namely employing statistical models to forecast mortality trends among women and high-risk age populations and analyzing these trends within healthcare systems through a 25-year nationwide comprehensive assessment, which together provide novel and robust evidence for designing targeted strategies to reduce mortality and advance health equity.

## 2 Materials and methods

### 2.1 Data source

This study adopted a retrospective observational study using mortality data from the CDC WONDER database, which provides publicly accessible mortality and population data based on US death certificates. We analyzed data from January 1, 1999, to December 31, 2023.

### 2.2 Study population and variables

The inclusion criteria were deaths of US residents aged 15 years or older occurring between 1999 and 2023, with alcohol-related neurological and psychiatric disorders listed as the underlying cause of death. These disorders were identified using International Classification of Diseases, 10th Revision (ICD-10) codes F10 (alcohol-induced mental and behavioral disorders), G31.2 (alcoholic neurodegeneration), and G62.1 (alcoholic polyneuropathy). The rationale behind the selection process is outlined below: F10 covers a wide range of alcohol-related psychopathology (e.g., dependence, intoxication, withdrawal) and is the core classification for mental disorders. G31.2 specifically marks chronic neurodegenerative disorders related to alcohol (e.g., Wernicke’s encephalopathy, Korsakoff’s psychosis), which represent typical central nervous system damage. G62.1 corresponds explicitly to alcoholic peripheral neuropathy. Exclusion criteria included deaths attributed to other neurological and psychiatric diseases, deaths outside the specified age range, records with mortality rates classified by the CDC as “unreliable” (based on a death count of fewer than 20, which fails to meet the National Center for Health Statistics’ reliability threshold) or “suppressed” (typically to protect privacy), and non-U. S. residents. Sex, geographic area, age group, and race/ethnicity (White, Black or African American, Hispanic, American Indian/Alaska Native, and Asian or Pacific Islander) were chosen and stratified. The location of death was also considered.

### 2.3 Ethical considerations

This study did not require institutional review board approval as we used publicly available de-identified datasets provided by the government. The study adhered to the reporting standards outlined in the Strengthening the Reporting of Observational Studies in Epidemiology (STROBE) guidelines (15).

### 2.4 Statistical methods

All data were analyzed using R (version 4.2.3). The crude mortality rate (CMR) and AAMR per 100,000 population were calculated for each population subgroup and year, with stratification by sex, census region, state, race/ethnicity, urbanization level, age group, and place of death. AAMR was determined by standardizing the death rates using the 2000 U. S. standard population. A linear regression model was employed to analyse the temporal trends in AAMR. The AAMR data was transformed using natural logarithms to satisfy the normality assumption for linear regression. Subsequently, a linear model was fitted with Year as the independent variable and the log-transformed AAMR as the dependent variable:


log(y_t)=β⋅+β₁t+ϵ_t

In this equation, β₁ represents the slope coefficient for the APC. APC was calculated as follows:


APC=(e(β1)−1)×100

The 95% confidence interval (CI) was calculated using the Delta method:


CI=(e(β₁±1.96×SE(β₁))(−1))×100

Where SE(β₁) is the standard error of the regression coefficient. APCs were compared using t-tests or one-way analysis of variance. All statistical tests were two-sided, and *p*-values < 0.05 were considered statistically significant.

To predict the future trend in AINP mortality rates in 2030, we adopted the autoregressive integrated moving average (ARIMA) (p,d,q) model, where p (autoregressive order), d (differencing order), and q (moving average order) were determined. Data was divided into training and validation sets primarily by the following principles: Firstly, it is necessary to capture key characteristics of baseline trend stability by leveraging the coverage range of the APC trend phase. Secondly, the validation set should be extended to encompass the 2020 pandemic outbreak phase, in order to evaluate the model’s ability to achieve high-precision forecasting under conditions of extreme volatility. The length of the training set must satisfy the ARIMA model’s requirement for sufficient sample size to identify long-term dependencies. Furthermore, a substantial volume of training material is necessary to capture stable baseline trend characteristics, thereby enabling comprehensive testing of the model’s generalized predictive capabilities in complex fluctuation scenarios. Data from 1999 to 2015 was used as the training set, and data from 2016 to 2023 for validation. The analyses focused on demographic groups including women and the high-risk group of middle-aged and older adults. Bayesian information criterion (BIC) and root mean square error (RMSE) were employed to evaluate model performance, with a lower BIC indicating a better trade-off between model fit and complexity (penalizing excessive parameters), and smaller RMSE reflecting lower prediction error.

## 3 Results

### 3.1 Overall and gender-specific trends

**Figure 1:**
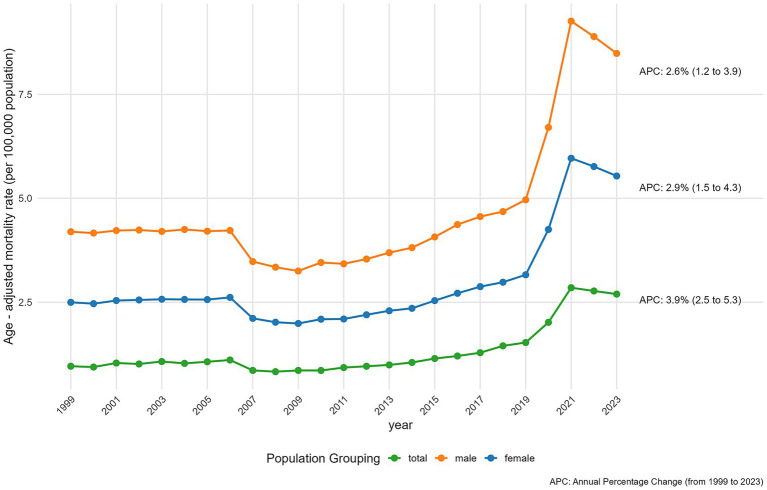
Overall and gender-specific AINP mortality rates in the United States from 1999 to 2023.

From 1999 to 2023, 226,785 people in the United States died from AINP. The overall AAMR increased from 2.28 in 1999 to 4.17 in 2023. Between 1999 and 2017, the AAMR showed no statistically significant temporal trend (APC = −0.04, 95% CI: −1.02 to 0.95; *p* = 0.934), meaning year-to-year fluctuations were consistent with random variation rather than a consistent increase or decrease. This was followed by a significant acceleration in increase from 2.84 to 4.51 between 2018 and 2021 (APC = 18.35, 95% CI: 11.19 to 25.98; *p* = 0.034). Subsequently, overall AAMR decreased from 4.51 in 2021 to 4.17 in 2023 (APC: -3.80, 95% CI: −4.33 to −3.27; *p* = 0.046) (Figure 1; Supplementary Figure 1).

As shown by CDC WONDER data, during the study period, AAMR for both female and male due to AINP in the United States exhibited a statistically significant increase. Specifically, for females, the APC in mortality was 3.89% (95% CI: 2.49 to 5.32; *p* < 0.001). Similarly, mortality rates for men also increased significantly, with an overall APC of 2.55% (95% CI: 1.23 to 3.89; *p* < 0.001).

### 3.2 AINP mortality trend based on geographic character

**Figure 2:**
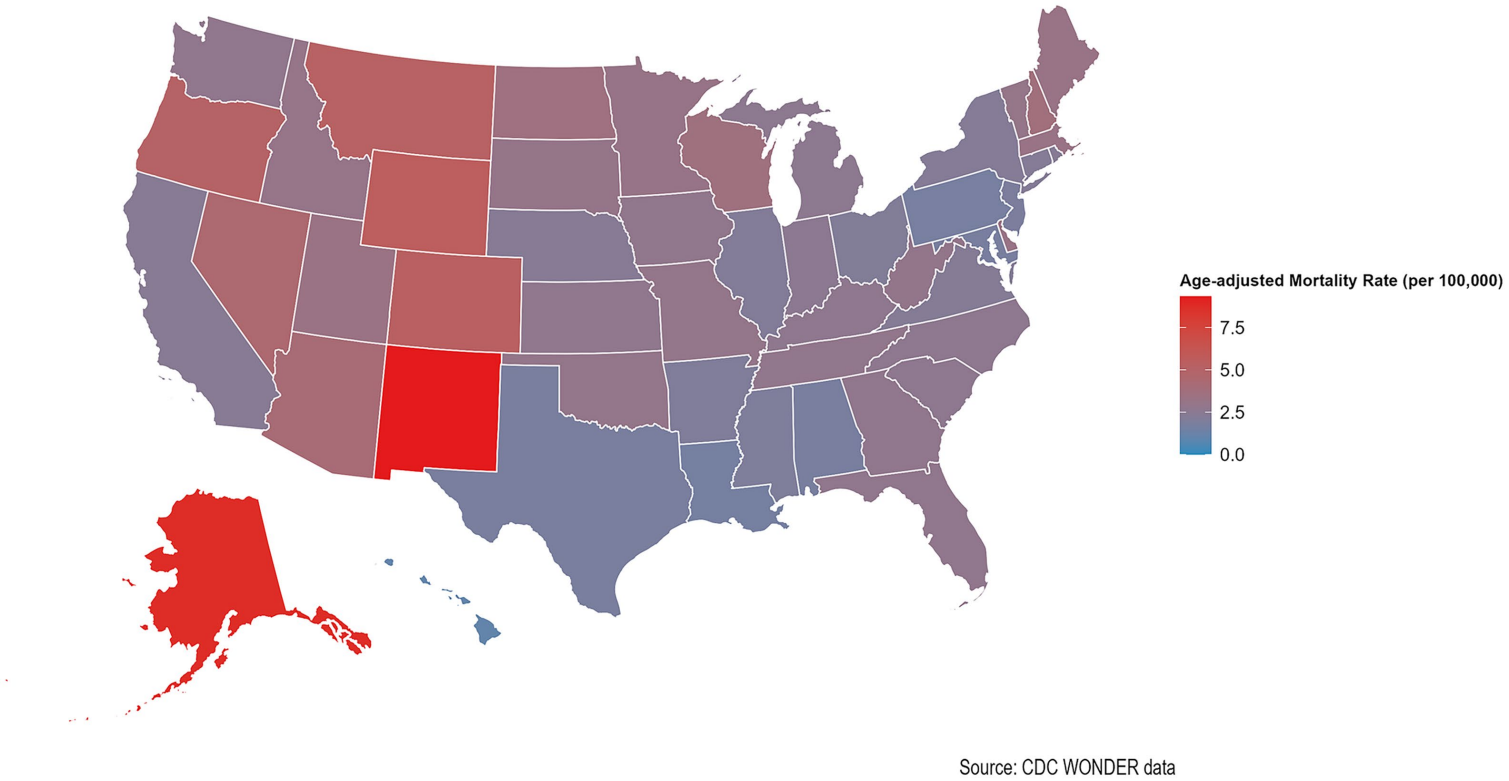
Geographic variation in AAMR due to AINP across the United States, 1999–2023. States are color-coded by their AAMR, using a gradient from blue to red, where darker shades of red indicate higher mortality rates. This static map was created using the usmap and ggplot2 packages within the R soft ware environment (version 4.2.3).

Significant differences in AINP mortality rates were observed across all states throughout the study period. From 1999 to 2023, the top five states with the highest AAMR were New Mexico, Alaska, Wyoming, Montana, and Oregon, while the bottom 10% included Hawaii, Louisiana, Texas, Maryland, and Alabama (Figure 2). Regarding APC changes, except for the District of Columbia, where the APC decreased significantly, the APC increased in 10 states (Alabama, Alaska, California, Georgia, Louisiana, Maryland, Mississippi, New Jersey, North Carolina, South Carolina) without reaching statistical significance, while the increase was statistically significant in the remaining 40 states (Supplementary Figure 2).

From 1999 to 2023, AAMR for AINP showed significant increases across all four U. S. Census regions, albeit with varying magnitudes. The Midwest region exhibited the most pronounced increase, followed by the Northeast region. The Western region demonstrated an intermediate increase, while the Southern region showed the most modest yet still statistically significant increase. Analysis by urbanization level revealed a clear gradient in mortality trends, with more urbanized areas generally demonstrating higher annual percentage increases. Large metropolitan areas showed the most substantial growth, with Large Fringe Metro areas exhibiting the highest APC, followed by Medium Metro areas and Large Central Metro areas. Small Metro areas demonstrated moderate growth, while nonmetropolitan areas showed divergent trends: Micropolitan areas exhibited significant increases, whereas NonCore areas showed no statistically significant change in mortality rates (Table 1).

**Table 1:** AMMR and APCs comparison among different region and urbanization.

Stratification	Group	Years	AAMR	APC (95% CI)	*P*-value
Region	Midwest	1999–2023	2.63	3.77% (2.43 to 5.12)	<0.001
	Northeast	1999–2023	2.26	2.52% (1.48 to 3.58)	<0.001
	South	1999–2023	2.38	1.80% (0.73 to 2.89)	0.0032
	West	1999–2023	3.36	2.08% (0.96 to 3.21)	0.0013
Urbanization	Large Central Metro	1999–2023	3.46	3.44% (1.20 to 5.73)	0.0059
	Large Fringe Metro	1999–2023	2.48	4.52% (2.53 to 6.55)	<0.001
	Medium Metro	1999–2023	3.15	4.24% (2.59 to 5.92)	<0.001
	Micropolitan (Nonmetro)	1999–2023	2.63	2.27% (1.38 to 3.17)	<0.001
	NonCore (Nonmetro)	1999–2023	2.34	0.49% (−0.50 to 1.49)	0.3409
	Small Metro	1999–2023	2.79	2.55% (1.62 to 3.49)	<0.001

### 3.3 Racial stratification

**Figure 3:**
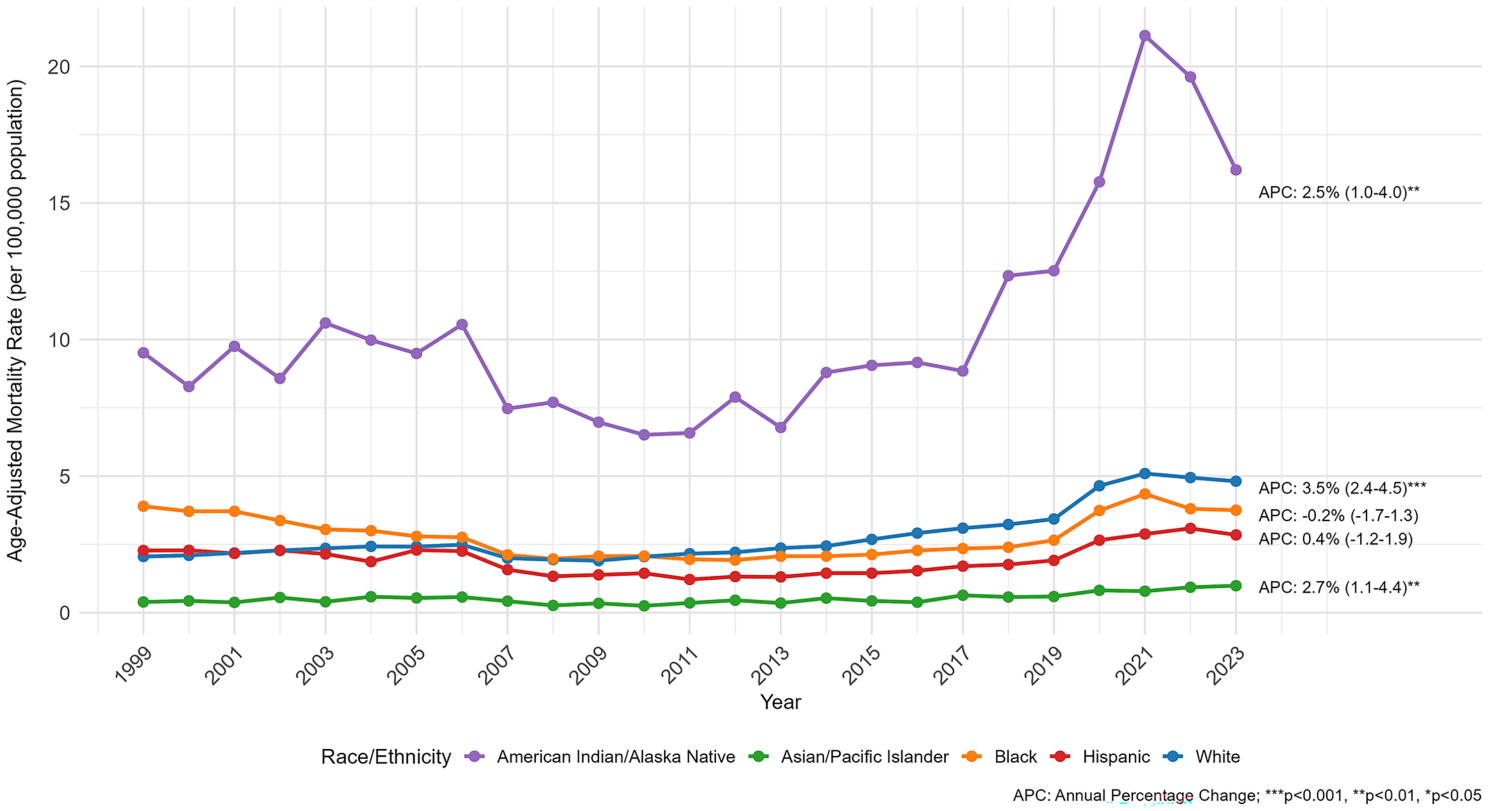
AINP mortality trends by race/ethnicity.

During the study period, American Indian/Alaska Native (AI/AN) populations showed the highest AAMR, with the lowest value of 6.51 in 2010 and the highest reaching 21.13 in 2021 (APC 2.50, 95% CI: 0.90 to 4.12%, *p* = 0.004). In terms of absolute burden, white people accounted for majority of deaths, totaling 170,014 (75.69%), and also showed a significant upward trend in AAMR (APC 3.45, 95% CI: 2.36 to 4.55%; *p* < 0.001). For Hispanic or Latino populations, the AAMR increased from 2.28 in 1999 to 2.85 in 2023 (APC 0.36%, *p* = 0.642), but this increase was not statistically significant. For Black or African American populations, the AAMR decreased from 3.90 in 1999 to 1.92 in 2012 (APC: −6.0% [95% CI: −6.8 to −5.1, *p* < 0.001]), and then increased to 3.75 in 2023 (APC: 7.7% [95% CI: 5.7 to 9.8, p < 0.001]). For Asian/Pacific Islander populations, the AAMR changed from 0.39 in 1999 to 0.99 in 2023 (APC: 2.74% [95% CI: 0.98–4.53%, *p* = 0.004]) (Figure 3).

### 3.4 Age-stratified analysis

**Figure 4:**
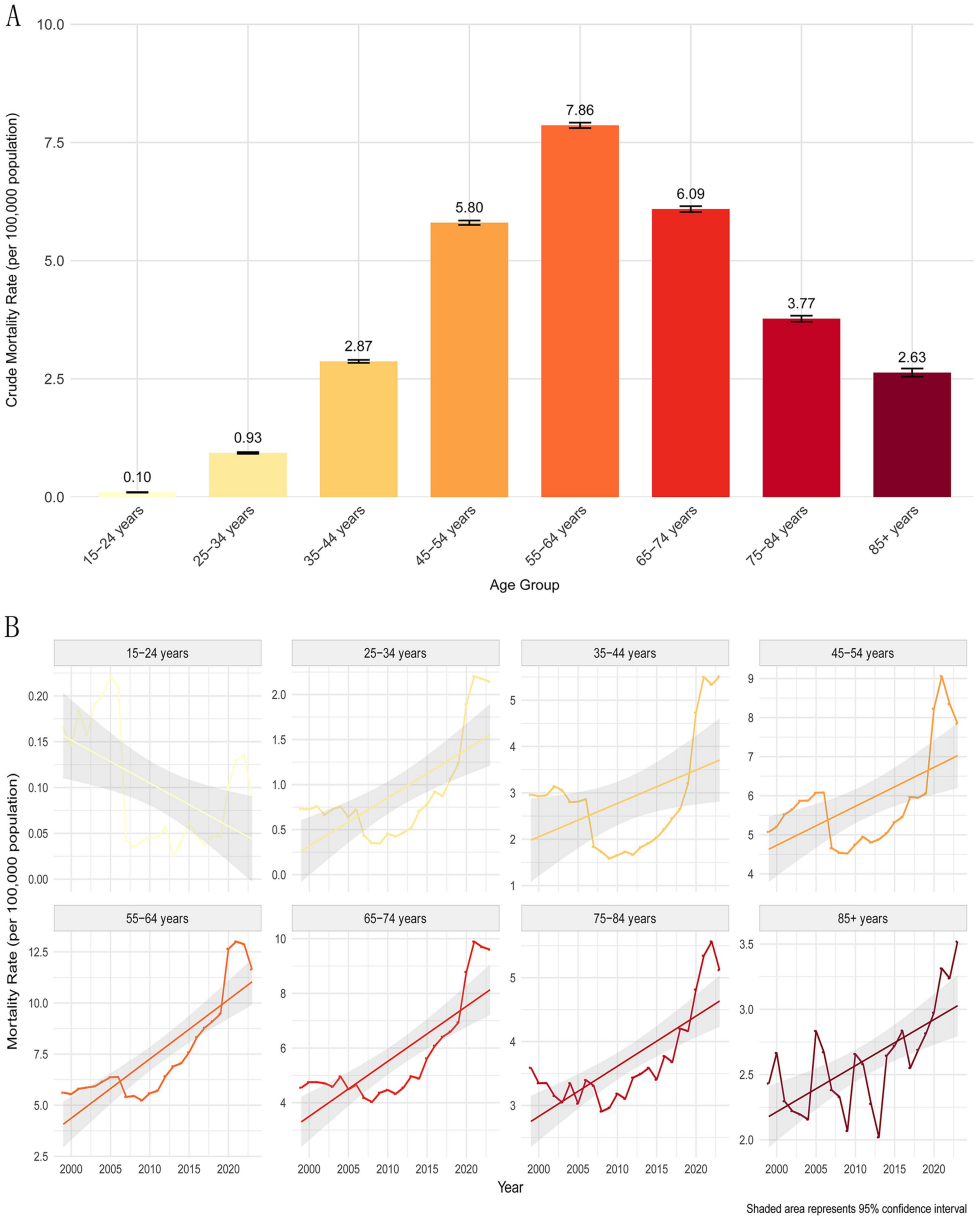
AINP mortality rates varied by age group. **(A)** Crude Mortality Rates by Age group (1999–2023) **(B)** Mortality trend of each age group. Shaded area represents 95% CI.

CMR of AINP varied according to age group, demonstrating an initial increase followed by a subsequent decrease with age, reaching its peak at 45–54 years and then gradually declining (Figure 4A). Adults aged 55–64 were most affected, accounting for 30.98% of all deaths (70,227 people), with a CMR of 7.86 per 100,000 (95% CI: 7.80–7.92), followed by those aged 45–54 (60,883 people, accounting for 26.86%) and those aged 65–74 (37,310 people, accounting for 16.46%). These data indicated that individuals aged 45–64, containing middle-aged and older adults, contributed to over 55% of the deaths. In contrast, the 15–24 age group was least affected (1,054 cases, accounting for 0.46%), and the crude mortality rate for those aged 85 and above was 2.63 per 100,000 (95% CI: 2.55 to 2.72). The absolute number of deaths among the older adults was relatively low, which may be attributed to survivor bias and a smaller population base (accounting for only 3.5% of the total population). With regard to the mortality trends according to age group, a statistically significant decrease in the rate was observed for the 15–24 age group. A non-significant increase was noted for the 35–44 age group, while a significant increase was recorded for all other age groups (Figure 4B).

### 3.5 Stratification of place of death

**Figure 5:**
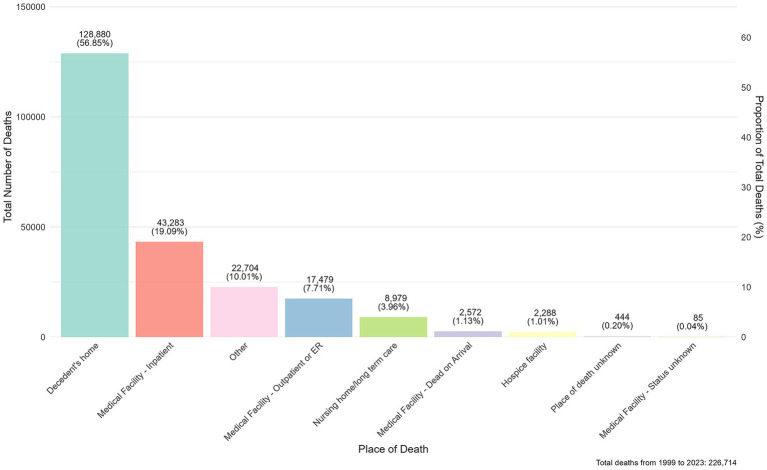
AINP mortality by place of death (1999–2023).

Data on the place of death for AINP patients showed that the majority of deaths occurred at the decedent’s home (56.85%), suggesting that these patients may not have had access to timely medical care services. The next most common location was Medical Facility–Inpatient (19.09%). There were 17,479 deaths (7.71%) among outpatients or emergency room patients. Deaths occurred in nursing home or long-term care facilities (3.96%), suggesting that these patients had chronic end-stage diseases. Other locations, including those categorized as “other” and “unknown,” accounted for only a small portion of deaths. There were 2,572 deaths (1.13%) upon arrival at the hospital, which may represent sudden unexpected deaths (Figure 5). Overall, the data indicate that most patients died at home, with a smaller proportion dying in medical facilities.

### 3.6 Projected CMR for high-risk populations by 2030

**Figure 6:**
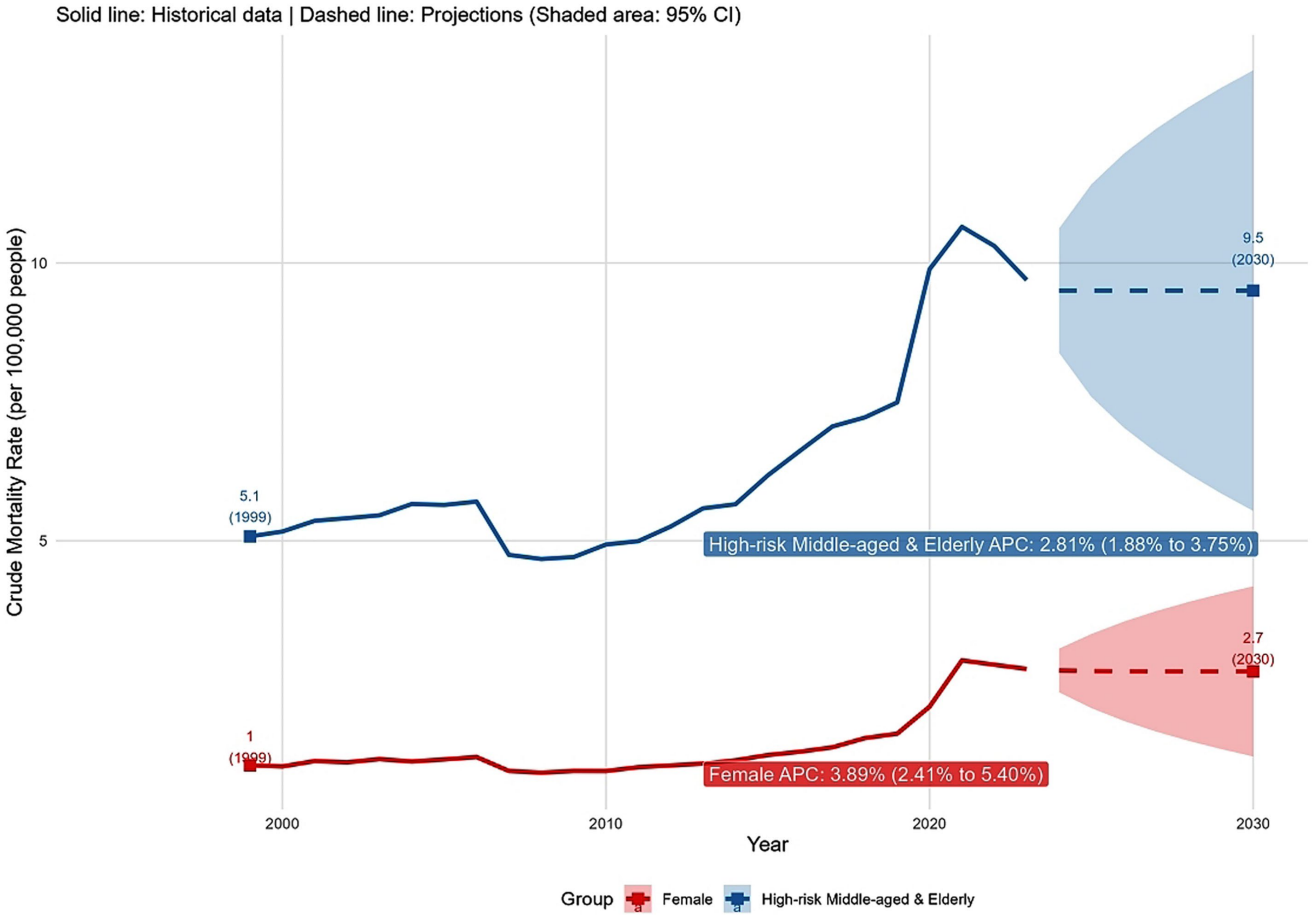
AINP CMR trends and projections (1999–2030) for female and middle aged and older adults.

The projected CMR for the female group was expected to increase to 2.70 in 2030, with an APC of 3.89% (95% CI: 2.41 to 5.40%) based on historical trends (Figure 6). The ARIMA model for this group showed a BIC of −37.1, a RMSE of 1.17, and ARIMA parameters (p, d, q) of (1, 0, 0), indicating the best fit among tested models. For the high-risk middle-aged and older adults (ages 45–74), the ARIMA model [BIC = 15.8, RMSE = 3.42, and parameters (p, d, q) of (1, 0, 0)] projected a slight decrease in CMR from 9.69 in 2023 to 9.50 in 2030. It is important to note that this projection represents the model estimate based on the entire time series structure, including autocorrelation. The positive APC of 2.81% (95% CI: 1.88 to 3.75%) calculated from historical data reflects the overall upward trend in the past data. However, the ARIMA forecast also incorporates recent fluctuations and the inherent serial correlation, which can result in short-to-medium-term forecasts that deviate from the long-term average trend, especially when the series exhibits volatility.

## 4 Discussion

### 4.1 Association between trends in AINP mortality rates and the COVID-19 pandemic

This study analysed mortality data from AINP among US drinkers, revealing its epidemiological trends and demographic distribution over the past two decades, thus providing crucial evidence for developing targeted intervention strategies (16). The findings indicated that the AAMR for AINP among the US population has shown a persistent upward trend over the past two decades, peaking temporally with the COVID-19 pandemic before declining slightly from 2021 to 2023. This temporal association was consistent with a report of a significant surge in alcohol-related deaths, particularly a striking increase in women (17). While a direct causal link cannot be established from our data, several pandemic-related factors likely contributed to this peak. The relaxation of alcohol policies, such as home delivery and to-go drinks, significantly enhanced alcohol availability and accessibility (18). Concurrently, a multitude of psychosocial stressors, including feelings of isolation, anxiety, depression, and financial uncertainty, motivated a considerable number of individuals to adopt alcohol consumption as a coping mechanism (18, 19). One report indicated that for every additional week of stay-at-home orders, the likelihood of binge drinking increased by 19% (19), suggesting a potential mechanism through which public health measures may have indirectly influenced risk behaviors. As alcohol is a known neurotoxin (10, 11) and a contributing factor to psychiatric disorders (12, 20), the confluence of heightened stress, increased consumption, and potentially strained healthcare access likely exacerbated underlying neuropsychiatric conditions, contributing to the observed rise in AINP mortality. Consequently, the period of the pandemic can be regarded as a complex interplay of factors that may have exerted a combined influence on the observed mortality peak.

### 4.2 AINP mortality rates vary significantly across gender, age, and ethnicity

The findings of this study indicated that AINP deaths occurred predominantly among males, although the APC for females was significantly higher than that for males. Males generally exhibited higher rates of alcohol consumption, and females demonstrated less efficient alcohol metabolism and were subject to elevated levels of psychological distress (21), which may more readily manifest as excessive drinking behaviors. As indicated by survey reports, a significant increase in the consumption of alcohol by women was also observed during the pandemic (17). Concurrently, the mortality rate among women has increased (22), resulting in a faster growth in their mortality.

Mortality rates demonstrated significant variation across different age groups. However, in contrast to the findings of some previous studies that reported elevated mortality rates from alcoholic hepatitis in younger populations (23), our study showed that AINP-related mortality rates were higher in middle-aged and older adults (45–74 years). The mortality rate remained highest in the 55–64 age group, with the steepest upward trend, which may be related to previous studies reporting that alcohol consumption among middle-aged and older adults increased at a significantly faster rate than among younger adults. Researchers found that between 2000 and 2016, the group with the largest increase in the prevalence of alcohol use and binge drinking was those aged 50 and older (24). Similarly, the rise in alcohol-related hospitalization rates among individuals aged 45–64 and 65 and over was more pronounced than that observed among younger adults (25).

The racial distribution of AINP mortality revealed critical disparities. First, with regard to the non-Hispanic White population, which constituted the predominant proportion of mortalities (75.6%), a substantial and sharply rising AAMR was observed over the study period. This indicates a considerable and increasing public health burden. These findings should be interpreted within the broader context of the “deaths of despair” epidemic, as described by Case and Deaton (26). This framework posits that premature mortality among middle-aged White Americans has been disproportionately driven by suicide, drug overdose, and alcohol-related causes, reflecting underlying socioeconomic distress and psychological despair. The elevated AINP mortality is consistent with the high population-level burden of alcohol use in this group, as evidenced by the recent NIAAA report which identified non-Hispanic White individuals as having the highest national prevalence of AUD (11.0%) (27). The high burden of AUD within this population may increase AINP prevalence, thereby elevating the risk of progression to fatal outcomes and contributing to higher mortality rates. This phenomenon may be attributed to a combination of factors, including a historically higher prevalence of alcohol dependence, sustained patterns of binge drinking, and sociobehavioral contexts that promote social drinking while these behaviours themselves are influenced by deeper socio-cultural factors (17, 28). This pattern was further corroborated by regional electronic health record data, which showed that White individuals had a higher AUD diagnosis rate in primary care compared to Black and Hispanic populations (29). This diagnostic disparity reflects actual racial differences in AUD prevalence and reinforces mortality disparities among AINP. Evidently, the disproportionately high mortality burden among white Americans results from the interplay of multidimensional factors. These factors are linked to socioeconomic hardships within the framework of ‘deaths of despair’, constrained by ethnic disparities in the burden of alcohol use, social behaviour patterns and the capacity of the healthcare system to identify the issue. The rising mortality among non-Hispanic white adults calls for a multi-level intervention. To tackle the root causes of despair, targeted socio-economic policies are needed, such as expanding employment support and accessible mental health services, to address underlying socioeconomic distress. Concurrently, integrating routine alcohol screening and evidence-based brief interventions into primary care may bridge diagnostic gaps and reduce AINP progression. This combined approach, linking upstream prevention (social policies) with downstream clinical care, is crucial to mitigating disparities.

In contrast, the most pronounced disparity was observed among non-Hispanic AI/AN individuals, who demonstrated the highest AAMR for AINP, significantly exceeding that of all other racial/ethnic groups. Despite the presence of known genetic variations in alcohol metabolism enzymes, such as alcohol dehydrogenase and aldehyde dehydrogenase, within this population (30), genetics alone is insufficient to explain the high rates of alcohol dependence. This finding indicates a multifaceted interplay of sociocultural and environmental factors driving the extreme burden in this community.

### 4.3 Regional distribution and policy implications

From a geographic perspective, 40 out of 54 U. S. states demonstrated increasing AINP mortality rates, with the most significant rise occurring in the Midwest. This phenomenon may be closely associated with the region’s alcohol policy environment, which was characterized by lax price controls and inadequate sales restrictions. It was also influenced by the sociocultural traits of the region, such as the prevalence of urban alcohol consumption cultures. Research indicates that a 10% increase in policy scores is associated with a 28% reduction in alcohol-related suicide rates (31). The absence of a coordinated policy framework for alcohol control across Midwestern states may be a pivotal factor contributing to the observed increase in mortality rates in this region.

Beyond regional variations, as indicated by the findings, mortality rates have been increased most significantly in urban areas, particularly in large fringe metropolitan areas. This pronounced trend emphasized the critical role of urban-specific factors in driving AINP mortality epidemic. The accelerated rise in these areas could be attributed to several urban-specific factors. The correlation between the density of liquor stores in a city and the level of alcohol consumption has been well-documented, with liquor stores often being found in less salubrious neighborhoods (32). Moreover, urbanization fostered alcohol consumption through dual mechanisms: “cultural permeation” characterized by the concentration of social venues and pervasive advertising, and “stress amplification” referring to fast-paced lifestyles and the heightened psychological pressure that accompanies them (33). Urban residents were more susceptible to increased alcohol use, frequently due to social demands and compounded by occupational stressors like workplace competition and economic anxiety. This was exacerbated in large fringe metros, where rapid growth, community dislocation, and weaker social cohesion may intensify stress and loneliness, leading individuals to use alcohol as a coping mechanism (34). Reports indicated that only about 26.1% of adults in the United States have received alcohol risk counseling from healthcare professionals (35). In large metro fringe areas where medical resources are often limited, counseling rates are likely even lower, resulting in at-risk individuals with AINP failing to receive timely and effective intervention.

### 4.4 Pace of death and structural barrier in AINP mortality

The finding that over 50% of AINP deaths occurred at the decedent’s home was a critical outcome that deserves examination through the perspective of structural health system barriers. This phenomenon likely reflects substantial barriers to healthcare access and utilization among people with AINP, rather than patient’s preference. It was reported that patients from socioeconomically deprived backgrounds often face substantial obstacles in obtaining consistent treatment (36). The “alcohol-harm paradox,” wherein disadvantaged groups experience greater alcohol-related harm despite similar or lower consumption levels, further highlights how systemic factors exacerbate these disparities. This was compounded by insurance-related disincentives. Alcohol Exclusion Laws, which permit insurers to deny coverage for injuries related to alcohol intoxication, remain active in 18 U. S. states as of 2023 (37). These laws create a structural stigma that deters individuals from seeking medical care and may discourage healthcare providers from conducting alcohol screenings due to reimbursement concerns. Furthermore, inequities in intervention were considered to contribute to the elevated rate of home deaths. Female and older adults, who were high-riks population for AINP mortality, received less alcohol related counseling, exacerbating the problem. To address the above issues, systemic change is necessary, including reforming harmful Alcohol Exclusion Laws, strengthening counseling and intervention in primary care, as well as optimizing the allocation of medical resource.

### 4.5 Predicting trends in vulnerable populations and public health implications

The present study employed the ARIMA model to forecast AINP mortality trends for the first time, with the potential to reduce human judgment errors (38). The findings suggested that the APC for females and the 45–74 age group will remain relatively stable, indicating that these two high-risk populations warrant future focus. This projection was consistent with long-term trends in alcohol use among middle-aged and older adults (24, 25) and shifts in female drinking patterns (22), while also providing clear direction for public health interventions. In light of the distinct risk profile across demographic groups, we propose targeted intervention for specific populations. For people aged 45–74, systematic alcohol screening should be implemented as part of routine geriatric health assessment and chronic disease management programs. Given their high mortality rate and frequent contact with the healthcare system for age-related conditions, such screening ought to be combined with brief interventions that address the unique risks of alcohol use in the context of polypharmacy, comorbid conditions, and life transitions such as retirement. For women, it is essential to develop gender-responsive interventions that address the psychological and social factors contributing to rising alcohol use. These may include integrating alcohol screening into mental health services, establishing support groups focused on stress, trauma, and social isolation, and training healthcare providers to better recognize the distinct presentations of AUD in women, who are more likely to engage in solitary drinking.

This study has several limitations. First, the analysis relies on death certificate data, which are subject to coding inaccuracies (e.g., in the attribution of the underlying cause of death) and potential underreporting of alcohol-related conditions, possibly leading to an underestimation of AINP mortality. Furthermore, as a retrospective analysis, it may be subject to unmeasured confounding variables. The absence of clinical trial data also prevents deeper insight into the causal relationships between specific AINP disorders and mortality. Lastly, unexamined heterogeneity-related biases could distort observed mortality trends. Future prospective research integrating clinical evidence is therefore necessary to investigate the multifactorial determinants of mortality in AINP, including genetic, environmental and socioeconomic variables.

## 5 Conclusion

The present study demonstrated an increasing trend in mortality rates associated with alcohol-induced neurological and psychiatric disorders. This finding emphasized the pressing need for targeted interventions and policy adjustments among high-risk populations geographic regions. The results of the study advocated for comprehensive public health strategies that required in-depth investigation into emerging risk factors such as comorbid conditions and drinking patterns.

## Data Availability

The datasets presented in this study can be found in online repositories. The names of the repository/repositories and accession number(s) can be found at: https://wonder.cdc.gov/deaths-by-underlying-cause.html.
