# Supplementary material for: The hidden epidemic of alcohol-induced neurological and psychiatric mortality in the U. S. (1999–2023): trends and disparities
Source: Front Public Health. 2026 Jan 8;13:1712253. doi: 10.3389/fpubh.2025.1712253 (PMC12823930; doi:10.3389/fpubh.2025.1712253)
Supplement: Supplementary file 3 [file Image_1.PDF]

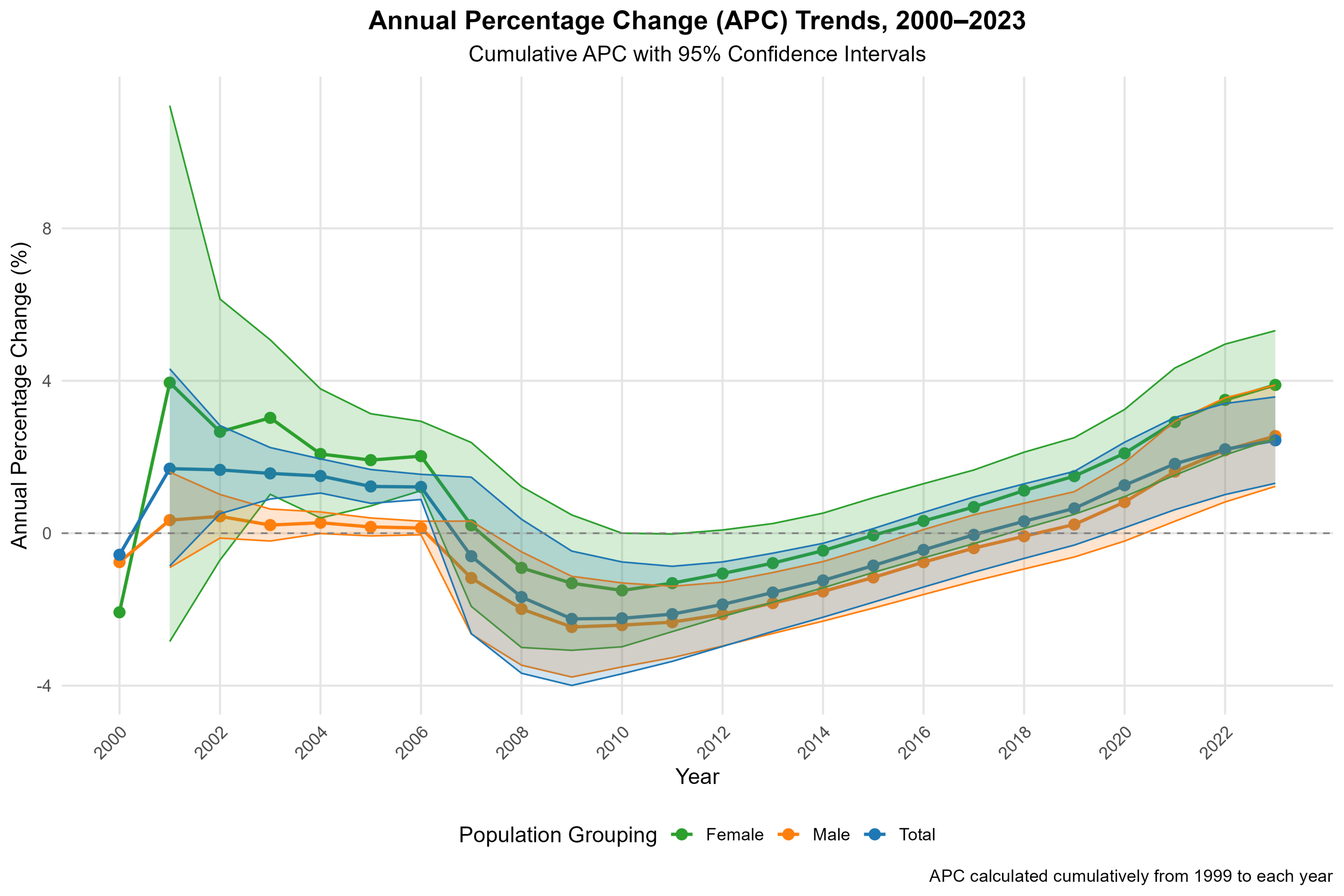
Annual Percentage Change (APC) Trends, 2000–2023
Cumulative APC with 95% Confidence Intervals
Annual Percentage Change (%)
8
4
0
-4
2000
2002
2004
2006
2008
2010
2012
2014
2016
2018
2020
2022
Year
Population Grouping
Female
Male
Total
APC calculated cumulatively from 1999 to each year
