# Supplementary figures and images for: The hidden epidemic of alcohol-induced neurological and psychiatric mortality in the U. S. (1999–2023): trends and disparities
Source: Front Public Health. 2026 Jan 8;13:1712253. doi: 10.3389/fpubh.2025.1712253 (PMC12823930; doi:10.3389/fpubh.2025.1712253)

State-level Trends in AINP-related Mortality Rate (1999-2023)

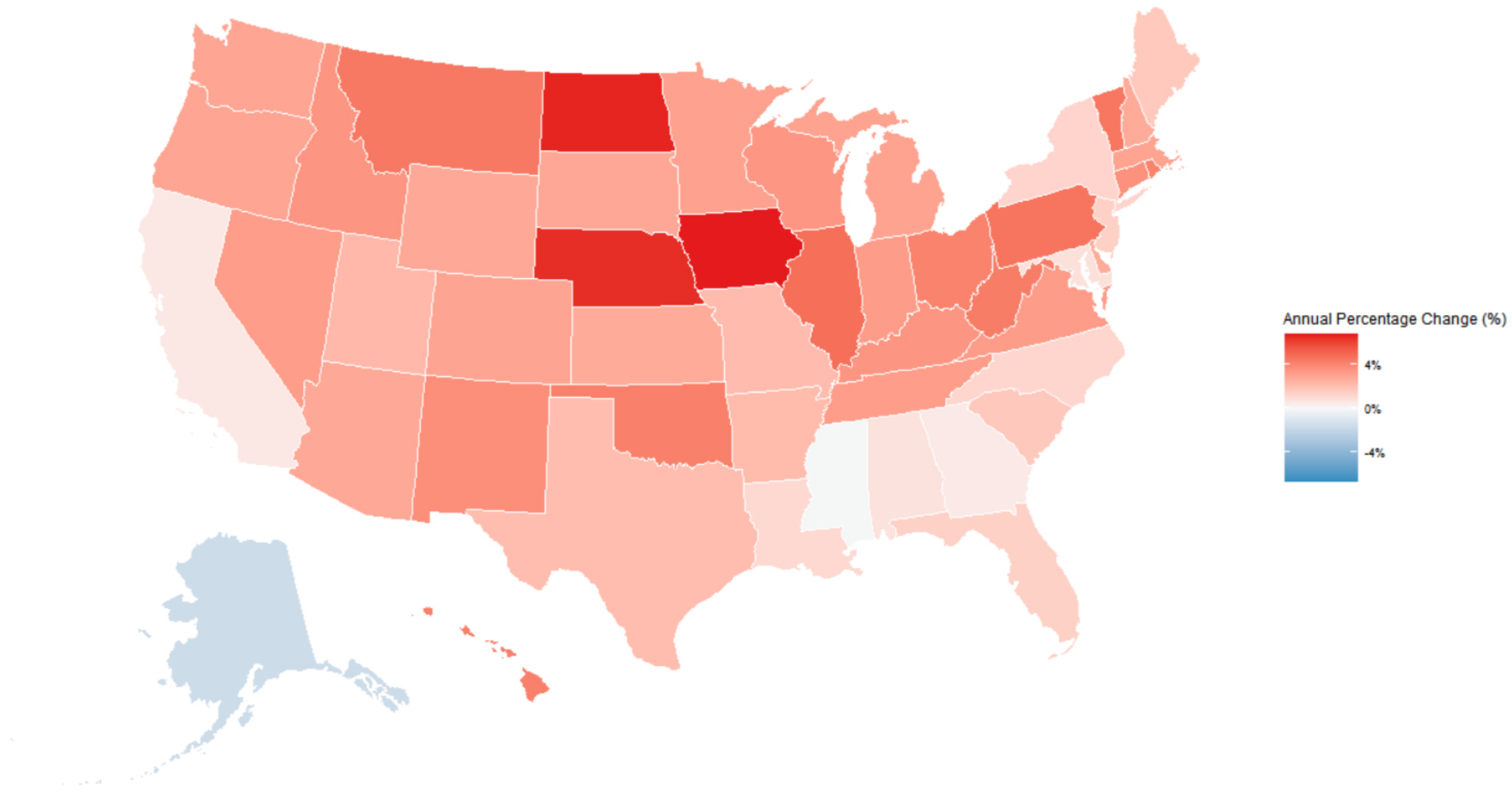

Supplement: Supplementary file 4 [file Image_2.PDF]

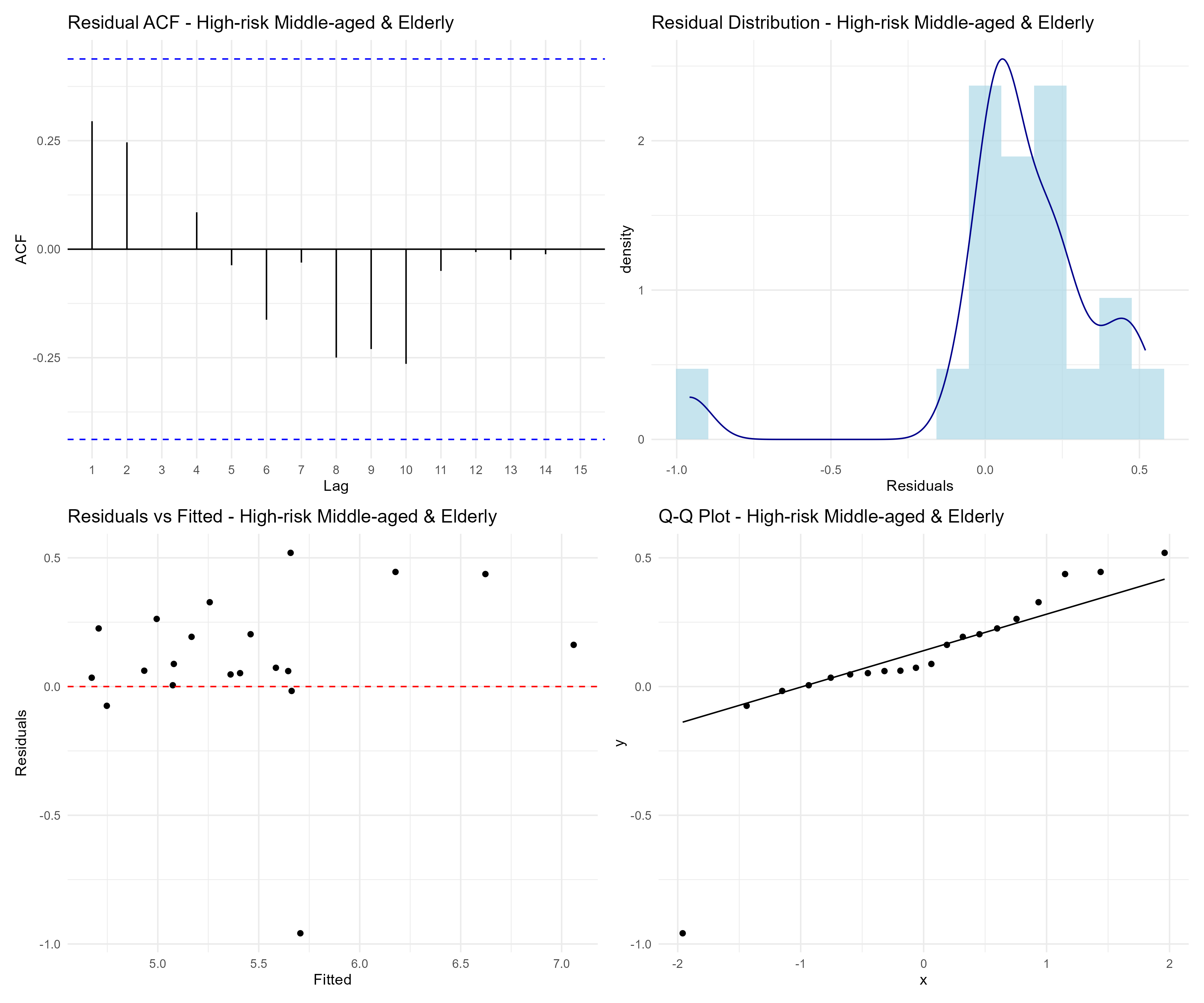

Supplement: Supplementary file 5 [file Image_3.PNG]

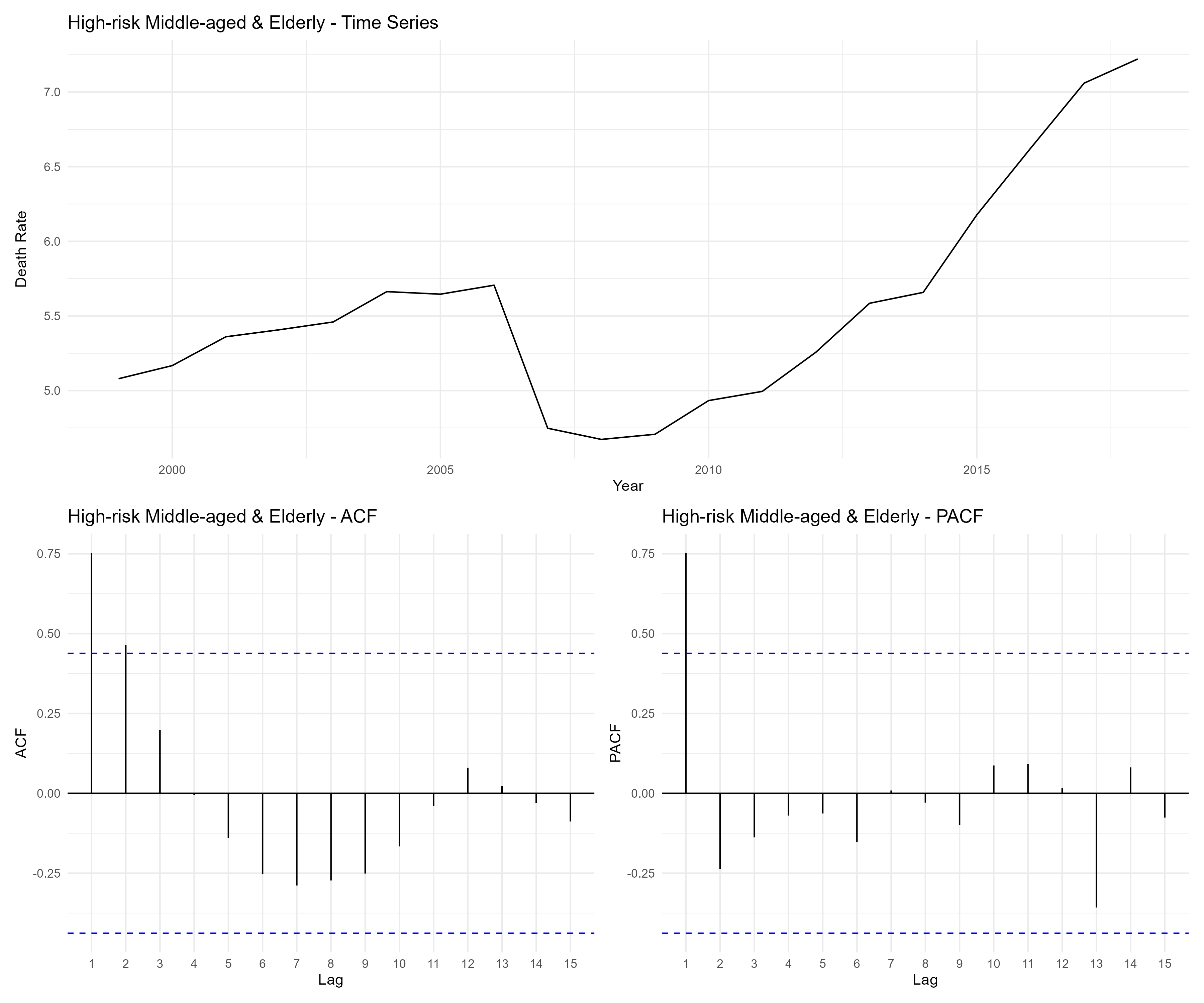

Supplement: Supplementary file 6 [file Image_4.PNG]

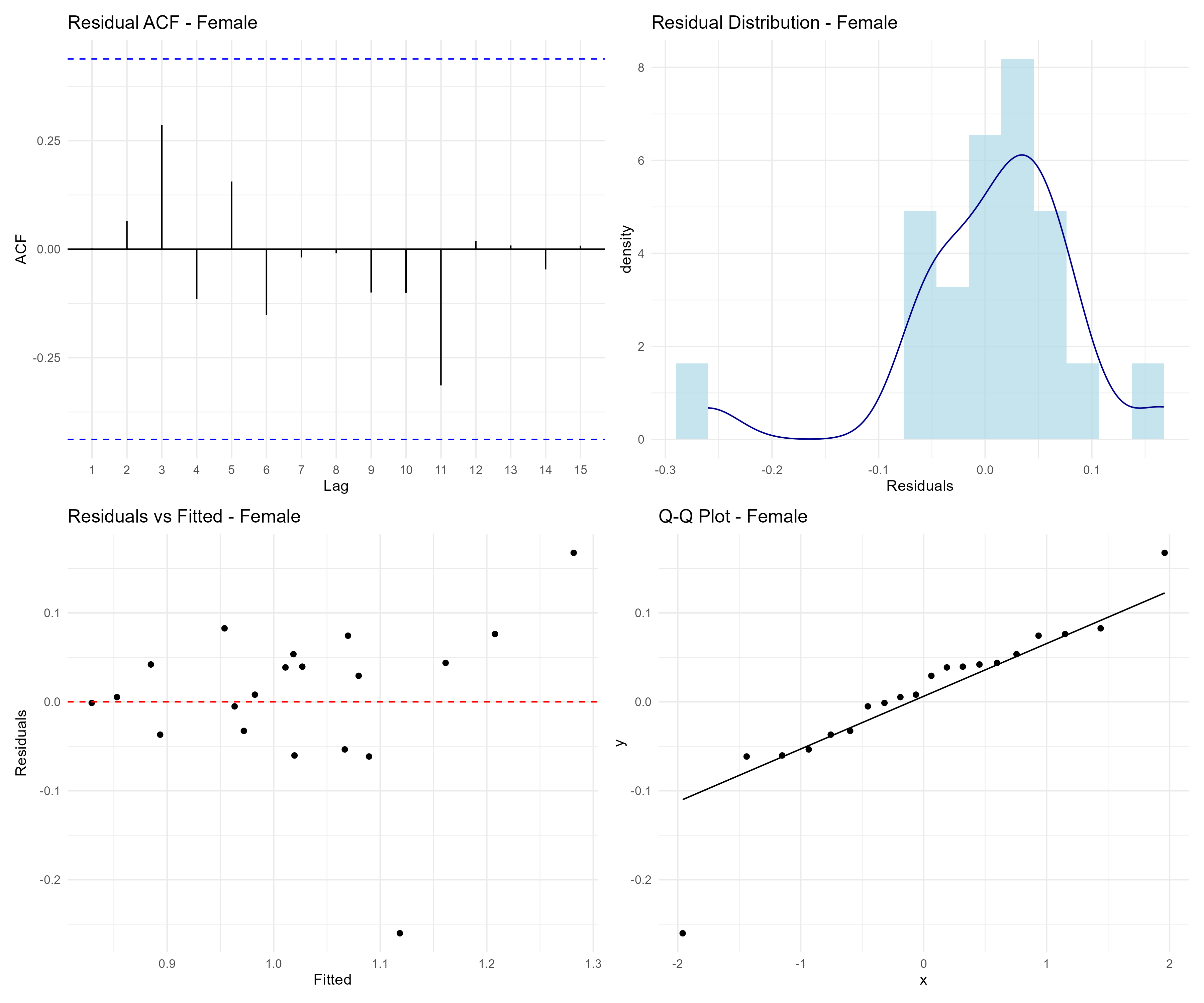

Supplement: Supplementary file 7 [file Image_5.PNG]

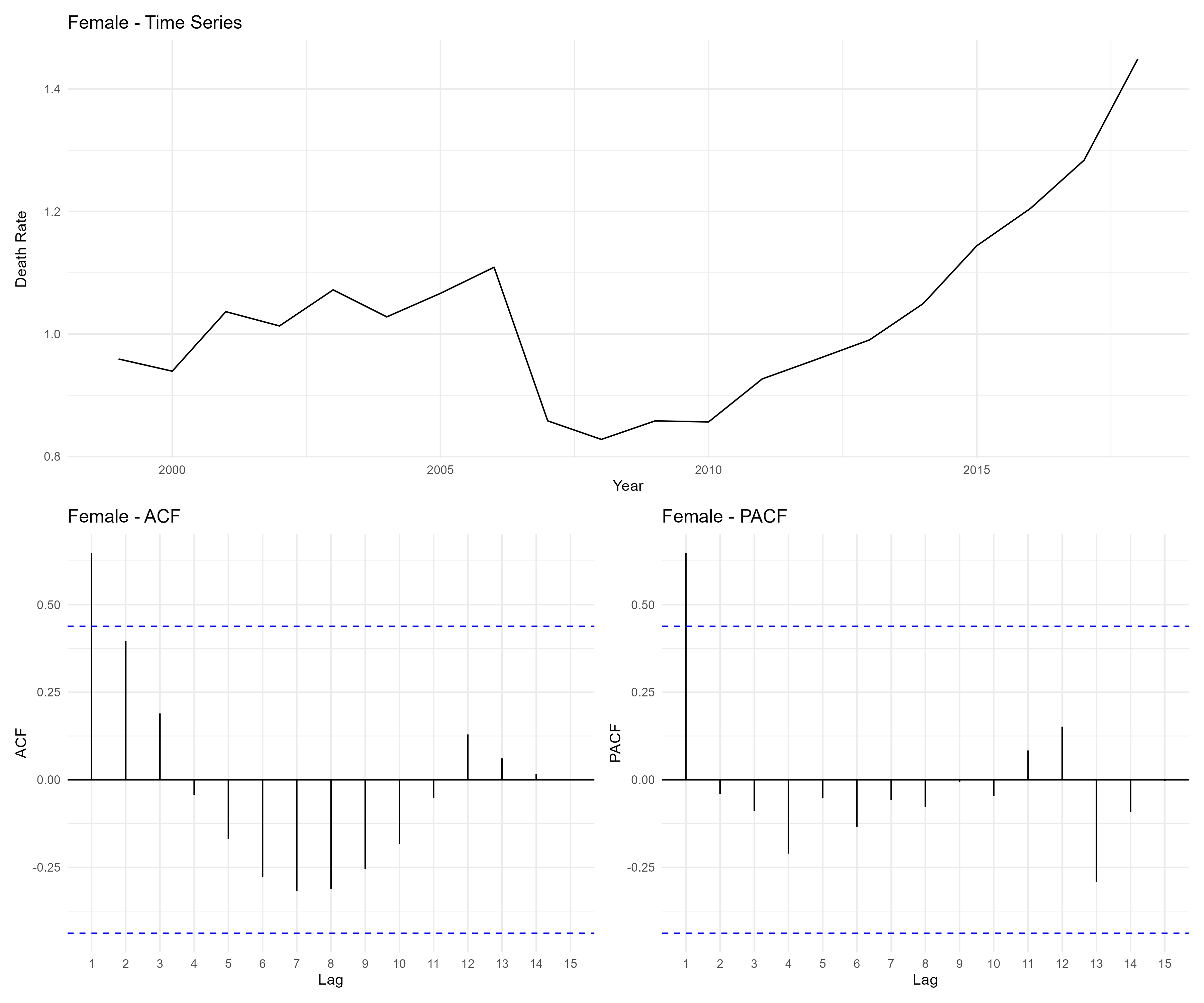

Supplement: Supplementary file 8 [file Image_6.PNG]
